# Surgical Pathology and Principles

**Published:** 1898-12

**Authors:** 


**Surgical Pathology and Principles.** By J. JACKSON CLARKE, M.B. Pp. xviii., 440. London: Longmans, Green, and Co. 1897.

In the introduction to this work the author points out that pathology, the science which treats of disease, includes the consideration of clinical phenomena as well as of the facts of morbid anatomy and etiology, and in the text many brief clinical descriptions of disease are given. We have ourselves found it distinctly advantageous from the practical point of view in teaching pathology, as in a museum class, to adopt a similar course. Of necessity, in a manual of the size of this, clinical description has to be somewhat meagre, the greater part of the text being devoted to morbid anatomy, macroscopical and microscopical, and to etiology. The value of the more strictly pathological portions of the work is enhanced by numerous illustrations, which are well described and cannot fail to be very helpful to the student.

In discussing diseases of the thyroid gland, the general enlargements other than the carcinomatous, sarcomatous, and exophthalmic varieties are described as being adenomatous, either nodose or diffused. We believe many of these enlargements are not the result of the development of adenomata in the gland, and prefer therefore to retain the terms simple goitre or hypertrophy of the thyroid for them, a nomenclature which the author has discarded.

In a paragraph on strangulated hernia it is stated that taxis, if successful, may cause an opportunity of doing a radical operation to be missed. We are not in accord with this way of stating the case. If taxis be successful in reducing the hernia, no opportunity for radical cure can be regarded as having previously existed which does not still exist. Taxis is the best mode of cure if it can be successfully applied, and is the mode which the majority of patients would certainly select if they fully understood the matter. The question of radical cure can be more judiciously considered by doctor and patient when the latter is not suffering from strangulation. The student should be cautioned that the existence of strangulation may tempt the surgeon to perform a radical operation in an unsuitable case, which is a serious error of practice.

The word flexuosity, used on page 340, is one we are not anxious to meet with again. A drawing to illustrate the structure of a cylindroma might, we think, give the student clearer ideas on the subject than does the description in the text on page 117.

